# Complete chloroplast genome of *Ligularia biceps* Kitam, 1941 (Asteraceae)

**DOI:** 10.1080/23802359.2022.2130716

**Published:** 2022-10-12

**Authors:** Yue-Yue Song, Chang-Neng Shi, Che Bian, Liang Xu, Yan-Yun Yang, Ming Xie, Ting-Guo Kang

**Affiliations:** School of Pharmacy, Liaoning University of Traditional Chinese Medicine, Dalian, China

**Keywords:** *Ligularia biceps*, chloroplast genome, phylogenetic tree, Asteraceae

## Abstract

*Ligularia biceps* is a plant belonging to *Ligularia* Cass., most of which have certain medicinal value. In this study, the chloroplast (cp) genome of *L. biceps* was sequenced for the first time. The *L. biceps* cp genome sequence length was 151,153 bp, with an large single-copy (LSC) region length of 83,259 bp, an small single-copy (SSC) region length of 18,234 bp, a pair of inverted repeat regions (IRs) length of 24,830 bp and GC content of 37.5%. In total, 131 genes were annotated, including 86 protein-coding genes, eight rRNA genes, and 37 tRNA genes. The phylogenetic tree was built based on 23 species, using the maximum-likelihood method. The results showed that the species clustered with other *Ligularia* Cass. species. This study provides a theoretical basis for establishing a classification system.

*Ligularia biceps* Kitam, 1941, is a perennial herb belonging to *Ligularia* Cass. of the tribe Senecioneae in the family Asteraceae. Most of the species in Northeast China are edible with medicinal and ornamental value. *Ligularia* Cass. plants generally have a long flowering period, and the flowers are primarily yellow. These species are usually planted in wetlands or forest margins. More than 130 species of *Ligularia* Cass have been described; only two are distributed in Europe and the remainder is distributed in Asia. The majority of *Ligularia* Cass. species are found in China, with 111 species and six varieties (Chinese Botany Committee 1993). *Ligularia* Cass. plants are typically distributed in temperate areas within a narrow range (Liu et al. 1994). The roots and stems of *L. fischeri* and *L. intermedia* are used in medicine, often in Tibetan medicine, Sichuan medicine, or other folk herbs, called ‘shanziwan’ and ‘guangziwan’, respectively (Jiang 1985; Zhou et al. 2019). ‘Shanziwan’ is commonly used in Northeast China and originates from the dry roots and rhizomes of *Ligularia fischeri* (Ledeb.) Turcz. This plant is primarily found in shady grasslands of the understory or in the forest margin (Zhao et al. 2011). The plant is rich in sesquiterpenoids, flavonoids, alkaloids, and other chemical components. Pharmacological studies have shown that this plant clears heat and detoxifies and is an antitussive expectorant with anti-inflammatory, antioxidant, and anti-tumor activities (Wang et al. 2012). In particular, the pharmacological effects of anti-tumor drugs have been thoroughly studied, and new anti-tumor drugs will be developed. In 2016, Li et al. found an unconfirmed *L. biceps* specimen on Baiyun Mountain in Fengcheng City, Liaoning Province. The plant was confirmed to be *L. biceps*, which is only sporadically distributed in Liaoning Province (Li et al. 2016). No study has reported *L. biceps* in China.

This study was approved by the School of Pharmacy of Liaoning University of Traditional Chinese Medicine in May 2018. All operations were carried out following the guidelines in the Specification on Good Agriculture and Collection Practices for Medicinal Plants (GACP; number: T/CCCMHPIE 2.1-2018). *L. biceps* is not a member of the national key protected wild plants, so collecting it is not against the Regulations of the People’s Republic of China on Wild Plant Protection. Article 5 of the regulations stipulates that the state encourages and supports scientific research on wild plants and on-site and *ex situ* protection of wild plants.

Whole-genome DNA was extracted from 150 mg samples of fresh leaves following a modified CTAB protocol (Doyle and Doyle 1987). The purified genomic DNA was sheared into approximately 350 bp fragments to construct a paired-end (PE) library following the Nextera XT sample preparation procedure (Illumina, San Diego, CA). The 150 bp PE reads were generated by a Novaseq 6000 sequencer (Illumina, San Diego, CA). The raw data totaled 4.69 Gb, and the clean data totaled 4.67 Gb after quality control processing using the NGS QC Tool Kit v2.3.3 software (Patel and Jain 2012). High-quality reads were assembled into the chloroplast (cp) genome using the *de novo* assembler SPAdes v.3.14.1 software (Bankevich et al. 2012). The PGA programme (Qu et al. 2019) was used to annotate the cp genome using the *Ligularia hodgsonii* (GenBank accession number MF539929) cp genome as a reference. The plant material used in the experiment was the fresh leaves of *L. biceps* collected on Baiyun Mountain, Fengcheng City, China (E 123°41′, N 40°23′), which was identified by Professor Liang Xu, Liaoning University of Traditional Chinese Medicine. The specimen was verified to be *L. biceps*, and the genomic DNA was preserved at the Key Laboratory of Traditional Chinese Medicine (Liang Xu 861364054@qq.com, *L. biceps* number: 10162220607868LY).

The entire circular genome was 151,153 bp in length, with a typical circular structure and GC contents of 37.5%. It contained a large single-copy region of 83,259 bp, a small single-copy region of 18,234 bp, and a pair of 24,830 bp long inverted repeat regions. It encoded 131 genes, including 86 protein-coding genes, eight rRNA genes, and 37 tRNA genes. The *rps16*, *atpF*, *rpoC1*, *petB*, *petD*, *rpl16*, *rpl2*, *ndhB*, and *tdhA* genes each contained one intron; the *clpP* and *ycf3* genes each contained two introns, and the *rps12* gene had trans-splicing.

Phylogenetic trees are widely used in genetic and evolutionary studies of various organisms. We selected the complete cp genome of 18 species (including *L. biceps*) to construct the phylogenetic tree, using the maximum-likelihood method with the model TVM + F+R4 in IQ-TREE 1.6.12 (Figure 1). The phylogenetic tree revealed that the species clustered with other plants in *Ligularia* Cass. and *Ginkgo biloba* was the outgroup, which was distant from the other species. This finding suggests that the results are authentic and reliable, laying a theoretical foundation for the systematic classification of Asteraceae plants and providing data to support molecular pharmacology.

**Figure 1.**
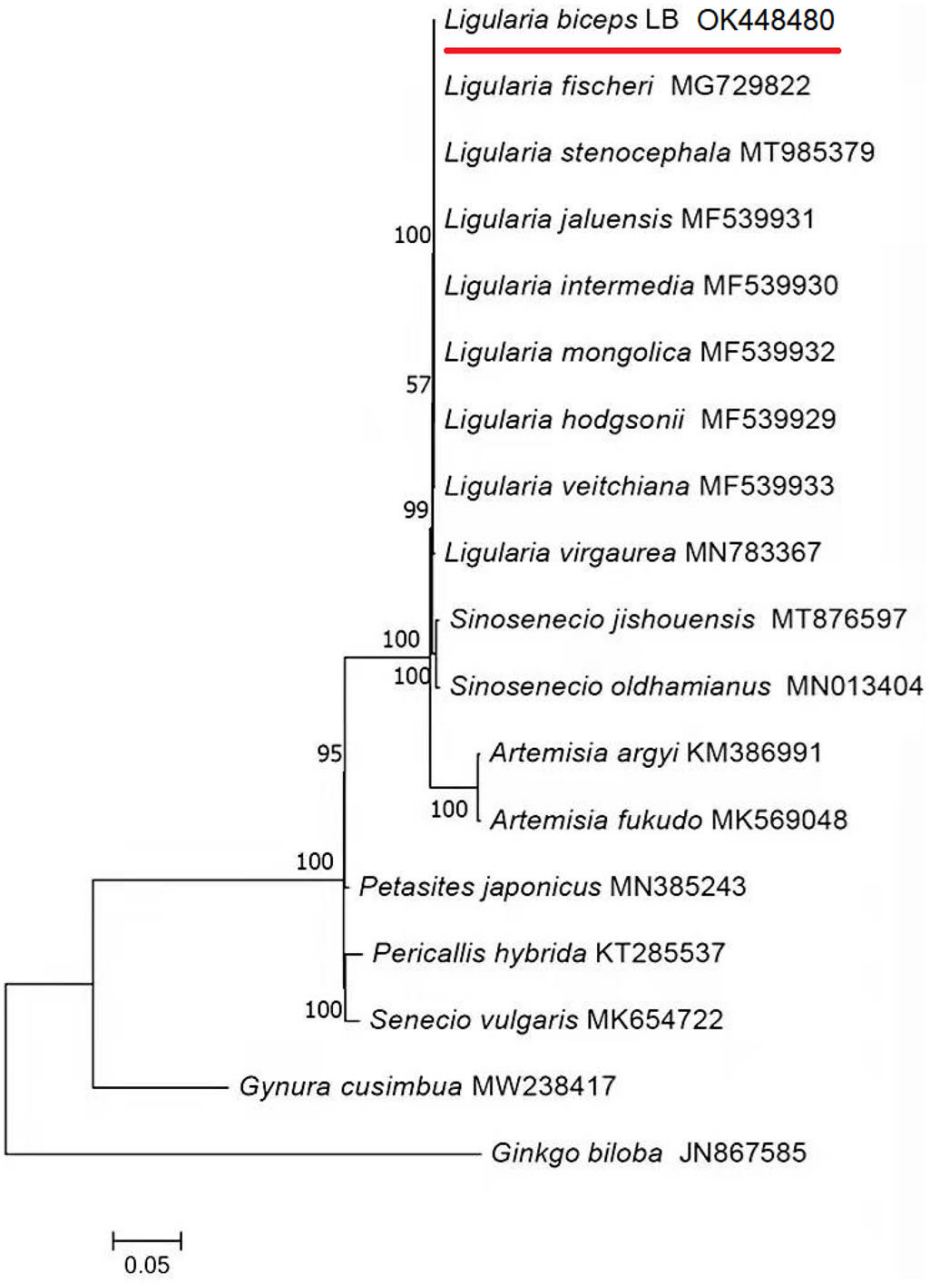
Maximum-likelihood (ML) phylogenetic tree of *L. biceps* and 17 other species. Numbers at nodes correspond to ML bootstrap percentages (1000 replicates).

## Data Availability

The genome sequence data that support the findings of this study are openly available in GenBank of NCBI at https://www.ncbi.nlm.nih.gov/ under the accession no. OK448480. The associated Bio Project, SRA, and Bio-Sample numbers are PRJNA769462, SRR16267454 (ILLUMINA), and SAMN22138609, respectively.
